# Preanalytical variables influencing the interpretation and reporting of biological tests on blood samples of living and deceased donors for human body materials

**DOI:** 10.1007/s10561-023-10106-z

**Published:** 2023-08-25

**Authors:** Elizaveta Padalko, Luc Colenbie, Alain Delforge, Nadine Ectors, Johan Guns, Romain Imbert, Hilde Jansens, Jean-Paul Pirnay, Marie-Pierre Rodenbach, Ivan Van Riet, Anne Vansteenbrugge, Gilbert Verbeken, Muriel Baltes, Hilde Beele

**Affiliations:** 1 Department of Medical Microbiology, Ghent University Hospital, C. Heymanslaan 10, 9000 Ghent, Belgium; 2 Department of Diagnostic Sciences, Ghent University, C. Heymanslaan 10, 9000 Ghent, Belgium; 3 Working Group on Cells, Tissues and Organs of the Superior Health Council, Brussels, Belgium; 4 Department of Transplant Center, Ghent University Hospital, C. Heymanslaan 10, 9000 Ghent, Belgium; 5 Laboratory of Clinical Cellular Therapy, Institute J. Bordet, Rue Meylemeersch 90, 1070 Brussels, Belgium; 6 Faculty of Medicine, KU Leuven (Catholic University of Leuven), Oude Markt 13, 3000 Leuven, Belgium; 7 Department of Laboratory Quality, Free University of Brussels VUB/University Hospital, Laarbeeklaan 101, 1090 Brussels, Belgium; 8 Department of Medically Assisted Reproduction, CHIREC, Boulevard du Triomphe 201, 1160 Brussels, Belgium; 9 Department of Medical Microbiology, Antwerp University/University Hospital, Drie Eikenstraat 655, 2650 Edegem, Belgium; 10 Laboratory for Molecular and Cellular Technology, Queen Astrid Military Hospital, Bruynstraat 1, B-1120 Brussels, Belgium; 11 Service du Sang, Croix-Rouge de Belgique, Rue du Fond du Maréchal 8, 5021 Suarlée, Belgium; 12 Department of Hematology, University Hospital Brussels (UZ Brussel), Jette, Belgium; 13 Department of Dermatology, Ghent University Hospital, C. Heymanslaan 10, 9000 Ghent, Belgium

**Keywords:** Preanalytical variables, Biological test, Donor of human body material, Deceased, Living, Post-mortem

## Abstract

With the present paper, the Working Group on Cells, Tissues and Organs and other experts of the Superior Health Council of Belgium aimed to provide stakeholders in material of human origin with advice on critical aspects of serological and nucleic acid test (NAT) testing, to improve virological safety of cell- and tissue and organ donation. The current paper focusses on a number of preanalytical variables which can be critical for any medical biology examination: (1) sampling related variables (type of samples, collection of the samples, volume of the sample, choice of specific tubes, identification of tubes), (2) variables related to transport, storage and processing of blood samples (transport, centrifugation and haemolysis, storage before and after centrifugation, use of serum versus plasma), (3) variables related to dilution (haemodilution, pooling of samples), and (4) test dependent variables (available tests and validation). Depending on the type of donor (deceased donor (heart-beating or non-heart beating) versus living donor (allogeneic, related, autologous), and the type of donated human material (cells, tissue or organs) additional factors can play a role: pre- and post-mortem sampling, conditions of sampling (e.g. morgue), haemodilution, possibility of retesting.

## Introduction

The Tissues and Cells Directive—2004/23/EC-, establishes the quality and safety standards for the donation, removal, acquisition, testing, processing, storage and distribution of human substances that must be met by the tissue establishments. The Tissues and Cells Directive—2004/23/EC—will shortly be replaced by a “Regulation” causing the actual national legislations to become obsolete. This Regulation will refer to the standards, safety and quality guidelines developed by the European Directorate for the Quality of Medicines & HealthCare (EDQM) and the European Centre for Disease Prevention and Control (ECDC).

The actual implementation of the 2004/23/EC within national legislations discusses performing the mandatory biological tests on samples from tissue donors. The tests have to be performed on donor serum or plasma and are divided into serological tests and NAT tests.

The goal of the performance of these tests is to prevent transmission of infectious diseases from donor to recipient or to anyone who comes into contact with the donor or the transplant material (e.g., tissue establishment and healthcare personnel). Depending on the results, human body material (HBM) is rejected or released (Theodoropoulos et al. 2018).

The following biological tests are mandatory: antibodies against and P24 antigen of-human immunodeficiency virus 1,2 (anti-HIV 1,2), antibodies against Hepatitis C virus (anti-HCV), Hepatitis B surface antigen (HBsAg), antibodies against Hepatitis B core antigen (anti-HBc), if relevant antibodies against Hepatitis B surface antigen (anti-HBs). For HBV, HCV and HIV screening of allogeneic donors, both serological and NAT testing of donor blood samples are needed. For syphilis, only serological tests (a treponemal screening test e.g. *T. pallidum* haemagglutination (TPHA) test, *T. pallidum* particle agglutination (TPPA) test, or treponemal enzyme immuno-assays (EIA) or chemiluminescence immuno-assays (CLIA)) need to be performed. In the case of autologous donors, NAT tests are not necessary (Kitchen et al. 2013).

However, no guidelines with regard to the pre-analytical errors within e.g. storage and maintenance conditions of the blood samples, nor the interpretation and reporting of viral serology and NAT testing are available.

The objective of the current article is to extensively discuss critical pre-analytical variables of mandatory biological tests for the different types of cell and tissue donors.

## Types of pre-analytical errors

Pre-analytical errors relate to multiple variables (Cudachar 2013; Guder 2012). Plebani et al. (2014) listed a number of pre-analytical errors linked to identification on the one hand and linked to the sample on the other hand:

- Preanalytical errors linked to sample information: unlabeled samples, mislabeled samples, insufficiently labeled samples, samples suspected to be from the wrong patient (“wrong blood in tube”), irregularities in transfusion labelling requirements (e.g. signature of phlebotomist), etc.
- Preanalytical errors linked to the sample: haemolyzed, clotted, icteric/lipemic, incorrect filling level/inadequate quantity, lost/not received, damaged during transportation, improperly stored, etc.

A recent paper of Sareen et al. (2017) confirms that the pre-analytical phase is an important component of total laboratory quality. Studies show that the preanalytical phase accounts for 46–68.2% of errors observed (Plebani et al. 2014).

In the current work the sampling step, and in particular the choice of sample tubes, the choice between serum and plasma, the donor's condition (deceased or living), and the timing of the sample in case of haemodilution (pre-transfusion or post-transfusion) and in case of deceased donors (pre-mortem or post-mortem) are highlighted initially.

Secondly, storage and transport of the samples to the analysis laboratory, the centrifugation of the blood samples, and the practice of sample pooling are discussed.

Finally, the importance of the standardisation of working methods and the choice of tests will be discussed, taking into account the differences in diagnostic sensitivity and specificity between tests and the availability of validated tests on the market.

All these various steps are essential. For optimally reliable results, it is important to limit variability and to take into account all interfering factors.

The requirements for tissues and cells may differ from those for organs, as different regulations apply. Organs can be procured from different types of deceased donors. Organ donation after Brainstem Death (DBD) is possible from patients whose death has been confirmed using neurological criteria (also known as brain-stem death or brain death). Organ donation after Circulatory Death (DCD), also known as donation after cardiac death or non-heartbeating donation, refers to patients whose death is confirmed using cardio-respiratory criteria. Tissues (e.g. cornea only or skin, cornea, bones and tendons, …—multi-tissue donation) and cells (e.g. pancreatic ß cells, liver cells, …) can be procured subsequently to organ donation. There are two principal types of DCD, controlled and uncontrolled (Maastricht classification according to Evrard 2014). There is considerable variation in the contributions that DCD, and even more the two types of DCD, make to deceased organ donation in different (European) countries. In contrast, tissues can be procured in uncontrolled DCD (e.g. death on arrival in emergency department) and even up to 24 h (48 h for skin and tympano-ossicular grafts) after circulatory stop if the maximal warm-ischaemia time (time between circulatory stop and cooling of the body) does not exceed 12 h. These donors are commonly described as “cold”donors, in contrast to “warm”donors. The purpose of these recommendations is to propose test conditions that offer quality and safety guarantees for any HBM (tissues, cells and organs) and to provide sufficient information to determine the level of risk of transmission. As donors may be multi-HBM-donors (including organs), it is appropriate to extend the routine test requirements for organ donation, to those for cells and tissue donation. Moreover, the national test requirements should at least be compatible with international, more specifically European Union requirements, in case the HBM is applied in another country.

The tests discussed in this work are not limited to serological tests, but relate to biological tests in general (e.g. also including NAT testing). Conversely, bacteriological tests are excluded.

The criteria for acceptance or rejection of tissues and cells based on those test results must be defined (standard operating procedures). The Responsible Person as regulated by article 17 of Directive 2004/23/EC will interpret the test results, based on an adequate knowledge of the accuracy of the infectious-disease test allowing a correct interpretation of the test results. Decisions of acceptance or rejection must comply with the regulations/legislations or, if there are not prescriptive, be based on standards of practice and/or risk assessments elaborated, for instance, by professional organisations, the ECDC, EDQM or the Substances of Human Origin (SoHO) department of the European Commission (EC). In the end, it always remains the responsibility of the transplant/implant physician to make a risk/benefit analysis based decision on rejection or acceptance of the HBM.

## Sampling

### Condition of the donor

#### Living donors

The living donors are patients so, there is a possibility to collect information on the donor's risk level through questioning and clinical examination and to obtain an additional sample at a later point in time if needed.

Blood collection of allogeneic living tissue donors is described in the 2004/23/EC and the national legislation (e.g. RD of 28 September 2009). In the case of non-reproductive HBM, the sample should be collected at the time of donation or within 7 days after the donation. For practical reasons blood sampling from allogeneic bone marrow and peripheral blood stem cell donors must be performed within 30 days prior to donation, before reaching a “clinical” point of no return for the recipient. Specifications of donation itself—partner donors or non-partner donors—will define the testing strategy. In a couple, serological tests are done before (≤ 3 months) the first donation. For further partner donations, additional blood samples must be obtained according to national legislation, but ≤ 24 months from the previous sampling. E.g. The Belgian legislation makes a further distinction between direct and indirect use. In the case of sperm and oocyte donation that is not within a couple, sperm donations must be quarantined for ≥ 180 days after the last procurement, after which repeat testing is required, but quarantine is not necessary if at each donation serology testing is combined with NAT for HIV, HBV and HCV (EDQM).

In the case of an autograft procedure, blood of the autologous donor needs to be collected within a month before transplantation or within 7 days after the donation (RD of 28 September 2009).

#### Deceased donors

Blood samples need to be taken within 48 h pre-mortem, or within 24 h post-mortem. In case of deceased donors, the possibility to collect an additional sample does not exist (EDQM, 2022; RD of 28 September 2009; Greenwald et al. 2018). With deceased neonatal donors biological testing may be performed on a blood sample of the donor's mother.

#### False negative/positive results due to the donor’s condition

During the first 6–12 months of life, a child’s immune system is not mature, therefore antibodies may not be produced against an infection, potentially generating a false-negative result. At a later age detection of antibodies against pathogens may be hampered in donors with immune-suppressive conditions or treatments, generating false negative results. Test results may also be invalidated by haemodilution (see 4.1.). False positive results could be detected from a transfer of “foreign” antibodies in the circulation of the donor (transplacental, transfusion).

### Collection of the samples

The 2006/17/CE and the EDQM do mention exceptional testing on other body fluids and secretions (these tests “must not be performed on other fluids or secretions such as the aqueous or vitreous humour unless specifically justified clinically using a validated test for such a fluid.”). Whole blood (to be processed into serum or plasma depending on the test used) is procured from the donor. Collection of the samples should follow general standards and regulations (e.g., Clinical Laboratory Standards Institute (CLSI) “Procedures for the Handling and Processing of Blood Specimens for Common Laboratory Tests: Approved Guideline”, 4th Edition, CLSI document GP44-A4, 2010). In order to differentiate from IV drug abuse, it is important to indicate the donor body site where the sample was taken, when a venous puncture is used for the blood collection.

### Volume of the sample

It is important to have a sufficient amount of blood in order to perform the necessary tests. In case of a deceased donor, there is no possibility to obtain a new sample in order to redo testing, e.g., if a technical problem has arisen in the laboratory.

In the case of a deceased multi-tissue donor, the various stakeholders (e.g., different procurement organisations, tissue establishments, etc.) need biological screening results. Sharing of results, especially in the context of interactions with other tissue establishments and with organisations such as Eurotransplant (in case of multi-tissue and organ donors) can help to reduce the amount of blood samples needed.

Therefore, it is important to define on beforehand in a mutual agreement with the lab not only the necessary volume and the priority of the tests to be performed, but also to mention the added value of sharing lab results.

### Choice of tubes

Depending on the tests, tubes with or without additives will be chosen in compliance with lab requirements. Biological tests for the detection of HIV, HBV, HCV and syphilis can be performed on serum or plasma. In most cases, serum is used for biological testing. A dry tube (without anticoagulant) or a tube with coagulation activator—a tube in which the blood coagulates—can be used. Gel separation vacutainers that separate plasma from red cells are particularly advantageous for NAT testing and preservation of virus titres. After centrifugation, the supernatant liquid obtained is the serum. NAT testing can be executed on ethylenediaminetetraacetic acid (EDTA) plasma or on serum. EDTA is an anticoagulant that inhibits blood and/or plasma from clotting, ensuring that the DNA or RNA to be detected is non-significantly changed prior to the analytical process. Anticoagulation occurs by binding calcium ions (Guder 2012). EDTA plasma is most often used for NAT testing.

### Pre-mortem sampling

If the sample is taken from a living, heart-beating donor, the blood quality, and more specifically with regard to the performance of the biological tests, is considered comparable to that from routine patient/blood donor samples.

### Post-mortem sampling

However, if the sample is retrieved from cadaveric, post-mortem, non-heart-beating donors, the blood quality, and more specifically with regard to the performance of the biological tests, cannot be considered comparable to that of routine patient samples, due to potential blood changes after death (e.g., haemolysis, autolysis, or blood contamination due to the appearance of substances in non-circulating blood originating from growth of micro-organisms, release of enzymes and products generated by autolysis, …) (Kitchen et al. 2013).

The EDQM guidelines state that in the case of a deceased donor, blood samples must have been obtained just before death or, if this was not possible, the time of sampling must be as soon as possible after death, and in any case within 24 h after death. However, a number of studies have demonstrated the potential for longer post-mortem interval times. Addressing the need for validation (for each individual test and kit) and acceptance by the national regulator is important. To verify the validity of NATs performed on blood specimens collected later than 24 h post- mortem, Meyer et al. (2012) monitored viral nucleic acid concentrations in blood samples of deceased patients who were infected with HIV (n = 7), HBV (n = 5), and HCV (n = 17). Samples were taken upon admission and at 12 h, 24 h, 36 h, and 48 h post-mortem. HIV and HCV RNA were quantified using a Cobas TaqMan device (Roche), while HBV DNA was quantified using an in-house PCR. A more than tenfold decrease of viral load was observed, in one HIV-infected patient only, in samples taken 36 h or 48 h post-mortem. For all other tested patients, the decrease of viral load in 36 h or 48 h post-mortem samples was less pronounced. Specimens of 3 HIV- and 2 HBV-infected patients taken 24 h post-mortem, or later, were even found to have increased concentrations (> tenfold), possibly due to post-mortem liberation of virus and/or its nucleic acid from particular cells or tissues. They concluded that their preliminary data indicate that the time point of blood collection for HIV, HBV and HCV testing using PCR may be extended to 36 h or even 48 h post-mortem, and thus improve availability of tissue donations (Meyer et al. 2012).

A more recent study was carried out by Schmack et al. (2020). Twenty paired ante- and post-mortem blood samples from cornea donors were obtained and subsequently analysed for hepatitis B surface antigen (HBsAg), hepatitis B antibody (anti-HBc), anti-HCV, HCV RNA, anti-HIV-1/2, and HIV p24 Ag using Abbott test systems. The sera were also spiked with reference materials in concentrations giving low and high positivity for HBV, HCV, and HIV markers. The spiked ante- and post-mortem sera from related donors showed similar results for HBsAg, anti-HBc, anti-HCV, HCV RNA, anti-HIV, and HIV p24 Ag, indicating a high stability of viral markers in cadaveric specimens. Three cornea donors had a medical history of HBV infection and revealed anti-HBc at similar levels in the ante- and post-mortem sera. In addition, there was a single post-mortem sample demonstrating a weak signal of anti-HIV-1 and HIV-1 p24 Ag. False-positive or false-negative results were not detected. The results obtained with the Abbott ARCHITECT analyzer and Abbott RealTime HCV PCR showed no significant differences. The authors concluded that the analysed screening assays are suitable for the detection of infectious markers of HBV, HCV, and HIV at similar levels in spiked ante- and post-mortem sera from cornea donors (Schmack et al. 2020).

### Labelling of tubes

This is a very crucial aspect. On the one hand, the labelling of the primary tube must correspond with the right donor. In addition there might be a second source of mistakes if there is a step of aliquoting into a secondary tube before transport to the laboratory. Using serum separator tubes with a barrier gel avoids transfer of serum to new tubes. At least two donor identifiers, such as the donor’s full name, date of birth and medical record number should be used. If other donor identification methods—e.g. a unique codification system—are used, it should be validated to ensure traceability.

## Variables related to transport, storage and processing of blood samples

The EU legislation does not provide clear guidelines concerning the storage and maintenance conditions of blood samples, although these variables may be of real importance in the context of donation of HBM, especially in the case of procurement at a different site than the laboratory site. In those cases, blood samples are obtained in a different hospital and might only be transported to the processing tissue establishment and/or the laboratory when the often elaborate and long-lasting HBM procurement procedures are finished. In agreement with the laboratory, that if responsible for specification of the transport and storage conditions, these elements must be included in the standard operating procedure, which describes in particular the conditions of transport, temperatures, temperature monitoring, duration, etc.

The blood samples will be centrifuged to separate the different components in the blood. It is important to make a distinction between the storage conditions before and after centrifugation (McCaughey et al. 2017).

Red blood cells (RBC) are one of the major blood components. Haemolysis indicates the disruption of the RBC membrane, which causes the release of haemoglobin and results in a discoloration of the sample after centrifugation. This forms a problem because (major) haemolysis can lead to false-positive test results, especially in serology and can cause false negative PCR results due to the inhibition of Taq polymerase. The temperature of the blood during storage before centrifugation is an important factor in the process of haemolysis. A temperature above 40 °C (e.g. environmental temperature during storage or transport) and below 1 °C (e.g. freezing before testing) causes haemolysis. Other factors that affect haemolysis are sampling technique (e.g., intravenous catheter versus venous puncture, sample transport, sample tube volume and filling) (McCaughey et al. 2017). Furthermore, haemolysis may also be caused by conditions related to the donor (e.g. infection, toxins, medication, autoimmune haemolytic anaemia, haemodialysis, haemolytic transfusion reaction).

After centrifugation, the plasma or serum (depending on the type of tube) does not contain RBC anymore. As there are lab-specific differences, it is important that the ranges of the storage conditions are mentioned explicitly in the convention between the tissue establishment and the lab. Transport and storage conditions prior to testing should be in accordance with the applicable rules for the safe transport of (risk-bearing) biological material. Companies provide a diagnostic test package insert with instructions concerning storage timing and temperature that must be followed. These inserts invariably state that the reliability of assay results cannot be guaranteed if there are any deviations from the instructions in the package insert.

This means that different timeframes between blood sampling and centrifugation, between centrifugation and initial storage (= transport conditions) and between initial and final storage can possibly influence the biological test results and the reliability of the test. Variation in storage temperature before centrifugation of the blood could also have an impact on the biological test results and the reliability and validity of the test.

Moreover, the recommendations of the companies in these inserts may differ in function of the use of plasma or serum, especially in the case of post-mortem samples. However, there are hardly any data concerning the impact of storage condition prior to centrifugation.

In the case of PCR screening for HBV, HCV and HIV, centrifugation should be executed within a certain time after blood collection according to the package inserts, in the rule within 6 h after sample collection. However, this is not always possible. Especially in the case of HBM procurement from a multi-tissue donor in a different hospital than the site of the tissue bank/or laboratory, it is sometimes not feasible to comply with the timeframes mentioned in the package inserts. Moreover, there are limited data in the scientific literature on whether different conditions before centrifugation could influence the biological test results and the reliability of the test, especially for these three NAT tests (Jarvis et al. 2002).

Storage conditions for samples after centrifugation seem to be less critical. Although many authors have reported that the storage conditions could affect RNA stability and, hence, HCV and HIV RNA detection, a number of studies have shown that storage after centrifugation does not have a major impact on test results. In 2003, José et al. studied HCV RNA stability in plasma samples after storage at different temperatures (−70, −20, 5 and 25 °C). Samples containing different HCV titers were stored and analyzed by qualitative or quantitative NAT techniques at defined time points. Samples containing high HCV RNA titers were stored at −20 °C and were followed-up during approximately 2.6–2.7 years, samples with intermediate concentrations during approximately 1 year and samples with 100 International Units/milliliter (IU/ml) during 2.5 years. Independently of the HCV RNA concentration, the results show absence of decay in HCV RNA detectability. Samples stored at 25 °C maintained their HCV RNA titer during 14 days and samples at 5 °C were stable for at least 3 months. José et al. 2003. Similar results were obtained for other viruses (José et al. 2005; Baleriola et al. 2011).

In 2017, Berger et al. measured the influence of storage time on EDTA plasma samples stored at 4 °C. As a general rule, refer to the manufacturer’s instructions.

Another problem is the freezing of samples before analysis. Some companies have validated their test for freezing, but in most cases only for a certain time and for certain ranges of temperature. In addition in the majority of cases, they do not indicate when after thawing the tests should be carried out or at what temperature the samples should be stored between the thawing procedure and the laboratory analysis. It is also important to validate if or how many freeze/thaw cycles on samples can be allowed. All these items should be discussed between the tissue establishment and the laboratory and be specified in the convention document between the parties concerned.

## Variables related to dilution

### Haemodilution

If transfusion or infusion of fluids (blood products, colloids and/or crystalloids) was performed shortly before donation, haemodilution may lead to a decreased detectability of the antibodies or antigens in the donor blood and possibly to false-negative results. An algorithm is applied to evaluate the degree of haemodilution (Kitchen et al. 2013). The EDQM states that it is current practice in a number of countries to consider 50% calculated haemodilution to be the maximum allowable to minimize the risk of a false-negative test result due to serum dilution.

Examples of the need of a haemodilution calculation include:

- *Ante-mortem* blood sample collection: if blood, blood components and/or colloids were administered in the 48 h preceding blood sampling, or if crystalloids were infused in the hour preceding blood sampling;
- *Post-mortem* blood sample collection: if blood, blood components and/or colloids were administered in the 48 h preceding death (circulatory arrest), or if crystalloids were infused in the hour preceding death (circulatory arrest).

We refer to EDQM for an example of a commonly used formula to assess the donor’s potential haemodilution, or plasma dilution, that can be applied when the donor received any fluids that may lead to haemodilution. Adaptations of the algorithms may be needed for body sizes outside the normal adult range. Allowances may need to be made for a very large or a very small adult donor, or a paediatric donor. In brief, a donor’s total plasma volume (TPV) and total blood volume (TBV) are estimated by calculations based on the donor’s body weight, then direct comparisons are made to amounts of recent transfusions and/or infusions that were administered before circulatory arrest or before collection of the blood sample, whichever occurs first:

- Estimate TPV of donor (weight in kg × 40 mL/kg; or, weight in kg ÷ 0.025);
- Estimate TBV of donor (weight in kg × 70 mL/kg; or, weight in kg ÷ 0.015);
- Calculate total blood (mL) received in the last 48 h (A);
- Calculate colloids (mL) received in last 48 h (B);
- Calculate crystalloids (mL) received in the last 1 h (C);
- Add B + C and compare to TPV (fluid volumes are compared);
- Add A + B + C and compare to TBV (mass/fluid volumes are compared);
- Does either comparison show > 50% dilution? if not, the blood sample qualifies and can be used for testing for infectious diseases.

Exceptionally, a tissue establishment may accept tissues and cells from a donor with plasma dilution of > 50%, but only if each required test has been validated appropriately for use with a diluted test specimen. In such cases, to help reduce risk, additional testing should also be performed using molecular tests (i.e. NAT) for HIV, HBC and HCV, and possibly for other viruses, depending on the donor’s travel history, underlying disease or other factors.

The collected blood can also be diluted if the sample is drawn in close proximity to an infusion or transfusion intravenous line, even if the donor is not actually haemodiluted or plasma-diluted. Samples should be drawn from the opposite side of the body in relation to the site of any infusion/transfusion.

Furthermore, in theory, a transfusion shortly before the donation and within diagnostic window of the tests applied can result in transmission of infectious agents to the donor.

### Pooling samples

Pooling of samples is common practice for NAT testing in blood establishments. EDQM guide provides guidelines for the detection limits against which the NAT tests must be validated and describes the aspects that are important when performing pooling.

The blood establishments work according to the instructions of the manufacturer, and rely mainly on the package leaflet of the manufacturer of their NAT equipment and diagnostic reagents.

They take care to achieve the minimum limit of detection (LOD) stipulated in the guideline, and make sure that it is achieved even if the samples are diluted (eg. 1/6).

Pooling of samples cannot be used in the field of cell- and tissue banking. In general, clinical diagnostic laboratories have to work on individual samples and that is also the case for molecular tests.

Pooling is only minimally discussed in the EDQM guide for tissues and cells, but the following statement is made for pooling of samples of the same donor: specimens of blood, serum or plasma from the same donor must not be mixed together for testing, whether collected at the same time or at a different time.

If tissue establishments outsource their biological testing to blood establishments, they should in accordance with national regulation stipulate in their agreement with the blood establishment that the serological, as well as the NAT testing has to be performed on strictly individual, not pooled, blood samples.

## Testing related variables

### Compliance

In EU member states, Annex II of Directive2006/17/EC, amended by Directive 2012/39/EU, specifies mandatory laboratory tests and general testing requirements for living and deceased donors of tissues and cells, and requires that any such laboratory and its tests must be accredited, designated, licensed and/or authorised by the competent authority.

### Validation of the tests

All assays used for donor testing within the EU should be CE-marked, and more recently compliant with the REGULATION (EU) 2017/746 OF THE EUROPEAN PARLIAMENT AND OF THE COUNCIL of 5 April 2017 on in vitro diagnostic medical devices and repealing Directive 98/79/EC and Commission Decision 2010/227/EU.

Many biological tests, including serological and molecular tests, used in the clinical laboratories are not specifically validated for the use on cadaveric blood samples.

Several diagnostic companies providing this type of tests have been heard by the working group of the SHC at two different occasions, once in the initial phase and once just before finalisation of the SHC advice (SHC 9525 publication date: May 2022). There was hardly any evolution in the availability between the start and the finalization of the advice (over 3–4 years—delayed due to COVID-19). This illustrates, the limited (expected) interest, from the companies, with regard to post-mortem tests (EDQM 2022).

When a clinical laboratory is performing tests on cadaveric blood samples, the lab should check if the particular assay is validated for use on cadaveric blood by the company that markets this assay. The validation of the test should be per parameter (e.g. HIV, HBV, HCV in case of molecular testing and anti-HIV 1,2; HBsAg, anti-HBs, anti-HBc, anti-HCV and syphilis serology separately) and per specimen type (e.g. EDTA plasma, serum) used.

Testing on other specimen types than blood-derived is in this particular setting not applicable. In case the test is validated for use on cadaveric blood samples for the sample type by the manufacturer of the assay, the verification procedure in the clinical laboratory should be in accordance with its quality system. It is responsibility of the clinical laboratory to use appropriate tests and implement them within existing quality system.

In case the test is not validated for use on cadaveric blood samples by the manufacturer of the assay, the clinical laboratory should perform a validation specifically for cadaveric blood samples. Examples of validation of techniques versus tests e.g. the Siemens-BEP-III automation system have also been published (Kalus et al. 2013).

The approach to verify or validate analytical tests will depend on whether test systems and kits, certified as compliant with the EU In Vitro Diagnostic (IVD) Medical Device Directive, are used or in-house developed analytical tests. In all cases, the validation plan should take into account the variety of sample types and materials to be tested, as there may be substances present that interfere.

Verification studies should be done to demonstrate that the performance of the IVD kit or test system, as used in the establishment for HBM, meets the expected specification. If using Pharmacopoeia methods, e.g. for sterility testing, the methods should be verified in accordance with the method monograph. For quantitative assays, the acceptance criteria should consider trueness (bias), precision, interferences, linearity, limits of detection, stability, and verification of the reference values of the own population. The uncertainty of measurement should be established and quoted with subsequent results. For qualitative tests, specificity and sensitivity are the key criteria.

In-house developed analytical tests must be validated. The acceptance criteria should consider trueness (bias), precision, analytical sensitivity, analytical specificity, linearity, stability, diagnostic specificity, diagnostic sensitivity and verification of the reference values of the own population.

## Agreement between tissue establishment and laboratory

As mentioned before, it is very important to have an agreement between the establishment for HBM and the laboratory that performs the serological and NAT testing, (regardless of whether the laboratory is internal to the institution or external to it). It may be useful to also involve the transplant centre in the agreement.

Because of the lack of unambiguous literature data and universal guidelines in this regard, and because of the major operational differences between the clinical labs that perform these tests, it is important that the content of such an agreement is discussed in detail with the concerned laboratory.

This agreement must include at least the following elements, but should not necessarily be limited to them:

- The type of cell or tissue donor (living vs deceased),
- The fact that it is often not possible to obtain a new sample,
- The minimal sample volume required,
- The priority of the tests to be performed, in the event of limited volume,
- The collection conditions (centrifugation, type of tubes, labelling (incl. anonymisation,…),
- The transport conditions (interval time, temperature, packaging, time of delivery),
- Information about the performed tests, their CE marking, their validation, frequency of performance, time to response, …,
- Performance of tests on individual samples, no pooling,
- Reporting and documentation (formatting) of the test results (Padalko et al. 2018; SHC 2016; e.g. results of microbiological cultures, etc.),
- The people who are responsible for selecting the tests to be performed,
- The licensing/certification/accreditation of the lab according to the relevant regulatory frame,
- The cost and the billing of the tests,
- The transmission of (as soon as possible in the event of positive) tests to the establishment of HBM and/or other involved stakeholders (e.g. transplant centre).

## Additional tests

Depending on factors like individual occupational/travel history and specific current or past clinical abnormalities of the donor as well as the epidemiological situation, the decision can be made to carry out other optional tests. These may include screening for (tropical) infections such as malaria, trypanosomiasis and viral infections such as West Nile virus and Zika virus. The need to perform such assays, or others, must be examined on a case-by-case basis. Scientific evidence for risk factors is provided by ECDC. Additional (preliminary) consultation of (specialized) testing laboratories may be advisable.

## Archived samples

Archived samples may be used for several purposes: look-back testing involving a new infectious agent, development of more accurate or new tests, or if investigating a report of a serious adverse reaction in a recipient of tissues or cells. If there are no national requirements, a risk-analysis-based decision should be performed to decide if, and for how long, archived samples must be retained.

## Conclusion

Pre-analytical variables refers to any and all procedures that occur during sample collection, prior to sample analysis. This involves patient identification, physical sample collection, sample transportation/storage to the testing site and sample preparation/conditioning. Pre-analytical errors account for 32–75% of laboratory errors (Bonini et al. 2002). The most common sources of error are related to: sample source (accurate identification of both patient and sample); processing methods (problems such as haemolysis, undue clotting in the blood tube, insufficient sample draw volume and modification of analyte concentration); data handling; wrong sample/label and sample handling. Since activities in the pre-analytical phase are neither performed entirely in the clinical laboratory nor under the control of laboratory personnel, they are harder to monitor and to improve from inside the performing clinical laboratory.

Considering the possible impact of the above mentioned preanalytical variables on the interpretation and reporting of the tests in donors of HBM, it is very important to address these issues in detail. Therefore, these are fundamental elements of a cell and tissue establishment’s quality system and should be discussed with the laboratory (ies) undertaking tissue-donor testing, especially when post-mortem samples are concerned. They should be described in detail in a service-level agreement (SLA) between the establishment for HBM and the laboratory that performs the serological and NAT testing. A list is provided with the elements that should minimally be included in this agreement.
